# Incidence and Predictors of Textbook Outcome after Minimally Invasive Esophagectomy for Cancer: A Two-Center Study

**DOI:** 10.3390/cancers16061109

**Published:** 2024-03-09

**Authors:** Evangelos Tagkalos, Peter Grimminger, Xing Gao, Chien-Hung Chiu, Eren Uzun, Hauke Lang, Yu-Wen Wen, Yin-Kai Chao

**Affiliations:** 1 Division of Thoracic Surgery, Chang Gung Memorial Hospital-Linkou, Chang Gung University, Taoyuan 33302, Taiwan; evangelos.tagkalos@unimedizin-mainz.de (E.T.); x.gao.1@erasmusmc.nl (X.G.); b9102067@cgmh.org.tw (C.-H.C.); 2 Clinic of General, Visceral and Transplant Surgery, University Medical Center Mainz, 55131 Mainz, Germany; peter.grimminger@unimedizin-mainz.de (P.G.); eren.uzun@unimedizin-mainz.de (E.U.); hauke.lang@unimedizin-mainz.de (H.L.); 3 Department of Biomedical Sciences, College of Medicine, Chang Gung University, Taoyuan 33302, Taiwan; ywwen@mail.cgu.edu.tw

**Keywords:** esophageal cancer, thoracoscopic esophagectomy, robotic esophagectomy, textbook outcome, survival

## Abstract

**Simple Summary:**

The textbook outcome (TBO), a multidimensional indicator that reflects an optimal perioperative course, has emerged as a significant prognostic variable in surgical oncology. Our study aimed to assess the occurrence and determinants of TBO following minimally invasive esophagectomy (MIE) for cancer. Within a cohort of 945 patients who underwent MIE at two high-volume centers, TBO was realized in 46.6% of cases, correlating with markedly better overall and disease-free survival. Upon conducting a multivariable analysis, we found that the use of RE (odds ratio (OR) = 1.527; 95% confidence interval (CI) = 1.149–2.028) was associated with a higher likelihood of achieving TBO, whereas a Charlson Comorbidity Index (CCI) of 2 or higher showed an opposite association (CCI2: OR = 0.687, 95% CI = 0.483–0.977; CCI ≥ 3: OR = 0.604, 95% CI = 0.399–0.915). The advantage of RE in attaining a higher rate of TBO, compared to VATE, remained statistically significant after applying inverse probability of treatment weighting, with rates of 53.3% for RE and 42.2% for VATE (*p* < 0.001).

**Abstract:**

Purpose: The textbook outcome (TBO), a multidimensional indicator that reflects an optimal perioperative course, has emerged as a significant prognostic variable in surgical oncology. Our study aimed to assess the occurrence and determinants of TBO following minimally invasive esophagectomy (MIE) for cancer. Methods: A total of 945 patients who had undergone MIE at two high-volume centers between 2008 and 2022 were analyzed. Multivariable logistic regression analysis was applied to identify the independent predictors of TBO. The potential selection bias associated with choosing between different MIE techniques—namely, robotic esophagectomy (RE) and video-assisted thoracoscopic esophagectomy (VATE)—was addressed by applying inverse probability of treatment weighting (IPTW). Results: TBO was realized in 46.6% of cases (*n* = 440), correlating with markedly better overall and disease-free survival. Multivariable analysis showed that treatment with RE (odds ratio (OR) = 1.527; 95% confidence interval (CI) = 1.149–2.028) was associated with a higher likelihood of achieving TBO, whereas a Charlson Comorbidity Index (CCI) of 2 or higher showed an opposite association (CCI2: OR = 0.687, 95% CI = 0.483–0.977; CCI ≥ 3: OR = 0.604, 95% CI = 0.399–0.915). The advantage of RE in attaining a higher rate of TBO, compared to VATE, remained statistically significant after applying IPTW, with rates of 53.3% for RE and 42.2% for VATE. Notably, RE contributed to a greater probability of thorough lymph node dissection, resection with negative margins, and the avoidance of major complications. Conclusion: TBO was realized in 46.6% of the patients who underwent MIE for cancer. Patients with a lower CCI and those who received RE were more likely to achieve TBO.

## 1. Introduction

In recent years, the treatment landscape for resectable esophageal cancer (EC) has continued to evolve from upfront surgery to the use neoadjuvant therapy [1,2]. Although the optimal strategy is still a matter of debate, the delivery of high-quality surgery is an essential component in the neoadjuvant setting. Several individual metrics—including the rates of R0 resection, the number of dissected lymph nodes, the rates of procedure-related complications, the number of unplanned readmissions, and short-term (30-day or 90-day) mortality rates—have been traditionally used to track surgical quality [3,4,5]. However, there is increasing evidence that combined outcome indicators may outperform single parameters for the clinical auditing of surgical quality. 

The concept of textbook outcome (TBO)—which was originally proposed in 2013 in the field of colorectal surgery [6]—has gained popularity as a multidimensional composite indicator reflecting an optimal perioperative course, including variables associated with radical resection, and an uneventful postoperative course. In different complex surgical procedures, including esophagectomy, an association between achieving TBO and more favorable survival outcomes has been described [7,8,9,10]. Unfortunately, TBO is generally realized by less than 40% of patients who have undergone esophagectomy for cancer [7,11,12].

In the pursuit of enhancing surgical outcomes, the minimally invasive approach to EC has been gaining traction [13,14]. Notably, an increasing body of evidence suggests that minimally invasive esophagectomy (MIE) may not only decrease perioperative morbidity, but also offer oncological outcomes that are at least on par with those of traditional open surgery for EC [15,16,17,18,19]. In addition, recent studies have highlighted promising TBO rates among patients with EC who have been treated with MIE [20]. However, the factors influencing TBO achievement in patients with EC undergoing MIE have remained elusive. 

To bridge this knowledge gap, we conducted a retrospective analysis utilizing data from two high-volume MIE centers. The primary objective of the current study was to determine the rate of TBO among patients undergoing MIE. The secondary aims were to identify the predictors of TBO, with a specific focus on comparing the established video-assisted thoracoscopic esophagectomy (VATE) technique and the emerging robotic esophagectomy (RE) approach.

## 2. Materials and Methods

### 2.1. Study Population and Inclusion/Exclusion Criteria

**Figure 1:**
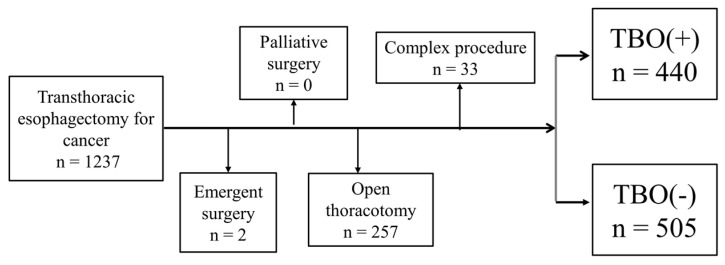
Study flowchart.

This study retrospectively analyzed prospectively gathered data from two high-volume tertiary referral centers: the Chang Gung Memorial Hospital-Linkou in Taiwan (referred to as Center B) and the University Medical Center Mainz in Germany (referred to as Center A). Patients with EC who underwent transthoracic esophagectomy were consecutively selected. The patient cohort from Taiwan was enrolled over a 14-year period between 2008 and 2022, whereas the German patient cohort was recruited over a 7-year period between 2015 and 2022. We excluded patients who underwent palliative surgery, required an open thoracotomy, or received complex esophageal resections such as esophagectomy combined with laryngectomy. Additionally, patients who underwent reconstruction methods other than gastric tube reconstruction were not eligible. The final analysis incorporated data from a total of 945 patients. Figure 1 provides a detailed study flowchart.

### 2.2. Neoadjuvant Therapy Protocol and Indication for Surgical Resection

In both centers, neoadjuvant chemoradiation (CRT) or chemotherapy (CT) followed by surgery were offered to patients with locally advanced esophageal malignancies located outside of the cervical area (i.e., cT2-4aNany or T1N+ when patients were deemed medically fit for surgery). The chemotherapy regimens administered for neoadjuvant CRT consisted of cisplatin plus 5-fluorouracil or carboplatin plus paclitaxel, both given concurrently with radiation therapy at doses ranging from 41.4 to 45 Gy. For neoadjuvant CT, the standard regimen was FLOT, which involves four preoperative and four postoperative two-week cycles of docetaxel (50 mg/m^2^), intravenous oxaliplatin (85 mg/m^2^), intravenous leucovorin (200 mg/m^2^), and fluorouracil (2600 mg/m^2^) administered over a 24 h period of continuous intravenous infusion. 

The standard surgical approach for esophageal malignancies located in the lower third consisted of a right transthoracic esophagectomy with intrathoracic gastric tube reconstruction (i.e., Ivor Lewis procedure). Neck anastomosis (i.e., McKeown procedure) was used for tumors of the cervical area or originating in upper two-thirds of the esophagus. In terms of the MIE technique, both RE and VATE were implemented across the two centers. The decision to proceed with RE or VATE in Center A was contingent upon the availability of a robotic system on the scheduled surgery date. Conversely, in Center B, all patients were offered RE as the primary option. However, if patients declined the partially insured robotic-assisted procedure, they were provided the alternative of undergoing VATE, which was fully covered by health insurance.

### 2.3. Definition of Variables

Comorbidities were defined using the Charlson comorbidity index (CCI) [21]. The criteria outlined by the Esophagectomy Complications Consensus Group (ECCG) were used to assess the occurrence of perioperative complications [22]. Complications were weighted according to severity based on the Clavien–Dindo classification (MCDC), with grades III–IV being considered as severe [23]. Circumferential resection margins were considered positive according to the criteria set forth by the College of American Pathologists (CAP).

### 2.4. Study Endpoints

The primary endpoint was the achievement of TBO—which was considered realized when the following criteria were simultaneously met: (1) no intraoperative complications (defined as any deviation from the ideal intraoperative course, such as the need for intraoperative transfusion, unintentional injury or resection of adjacent organs, and the necessity to switch to open surgery from a minimally invasive approach), (2) margin-negative resections, (3) lymph node yield ≥ 15, (4) no severe postoperative complications, (5) no need for re-intervention, (6) no readmissions to an intensive care unit (ICU), (7) length of hospital stay ≤ 21 days, (8) no 90-day postoperative mortality, and (9) no readmissions within the first 30 days from discharge. Overall survival (OS) was defined as the time from the date of surgery to the last follow-up visit or death from any cause. Disease-free survival (DFS) was measured from the date of surgery to the date of second cancer, locoregional recurrence, distant metastases, or death from any cause, whichever occurred first. Follow-up was terminated on 30 November 2022.

### 2.5. Statistical Analysis

Normally distributed continuous variables are here expressed as means ± standard deviations (SDs). Continuous variables with a skewed distribution are presented as medians and interquartile ranges (IQRs), whereas categorical data are given as counts and frequencies. The Student’s *t*-test and the Mann–Whitney U test were used to compare normally distributed and skewed continuous data, respectively. Categorical variables were analyzed with the chi-squared test. Univariate and multivariable logistic regression analyses were applied to assess the associations of clinicopathologic parameters with TBO. Variables entered in the univariate logistic regression analysis included previously described predictors of TBO, known risk factors for postoperative morbidity, and the main predictor of interest (i.e., the technique used for MIE). A multivariable backward selection procedure was implemented, with a threshold *p* < 0.1 for inclusion and *p* < 0.05 being defined as statistically significant in the final model. For each variable, the odds ratio (OR) and the associated 95% confidence intervals (CIs) were computed. To mitigate potential selection bias between RE and VATE, inverse probability of treatment weighting (IPTW) was employed. The propensity score (PS), derived from a logistic regression model, quantified the likelihood of patients undergoing either RE or VATE based on their observed baseline characteristics [24,25]. This model incorporated variables such as age, sex, body mass index (BMI), smoking history, CCI, type of tumor histology, clinical stage, utilization of preoperative therapy, type of resection (McKeown or Ivor Lewis), abdominal procedure type, and the center providing treatment. Inverse probability weights, calculated from the PS, facilitated the creation of a pseudopopulation. Specifically, for the RE group, weights were assigned inversely proportional to the PS, whereas for the VATE group, weights were inversely proportional to 1 minus the PS. This approach, utilizing IPTW, ensured the generation of stabilized weights, thereby preserving the integrity of the matched sample. The balance of covariates was evaluated using the standardized mean difference (SMD) both before and after the application of IPTW, with an SMD below 0.1 indicating an acceptable level of balance. Survival outcomes, including overall survival (OS) and disease-free survival (DFS), were visualized using Kaplan–Meier curves, with statistical differences assessed via the log-rank test. Data were analyzed using SPSS, version 25.0 (IBM Corp., Armonk, NY, USA), and R version 4.3.1 (R Foundation for Statistical Computing, Vienna, Austria). All statistical tests were two-sided, and a *p* value < 0.05 was considered statistically significant.

## 3. Results

### 3.1. Study Patients

The study included 945 patients, with 426 from Center A and 519 from Center B. Among them, 440 (46.6%) achieved TBO, while 505 (53.4%) did not. The general characteristics of the two groups are summarized in Table 1. The TBO group had (1) a higher BMI, (2) a lower CCI, and (3) a higher prevalence of adenocarcinoma than the no-TBO group. Conversely, patients who did not achieve TBO showed a higher frequency of the following variables: (1) preoperative radiotherapy, (2) use of VATE for the thoracic phase, (3) use of open laparotomy for the abdominal phase, and (4) use of the McKeown procedure. Significant differences with respect to TBO rates were observed between the two study centers (*p* = 0.001).

#### 3.1.1. Survival Outcomes in Relation to the Achievement of TBO

**Figure 2:**
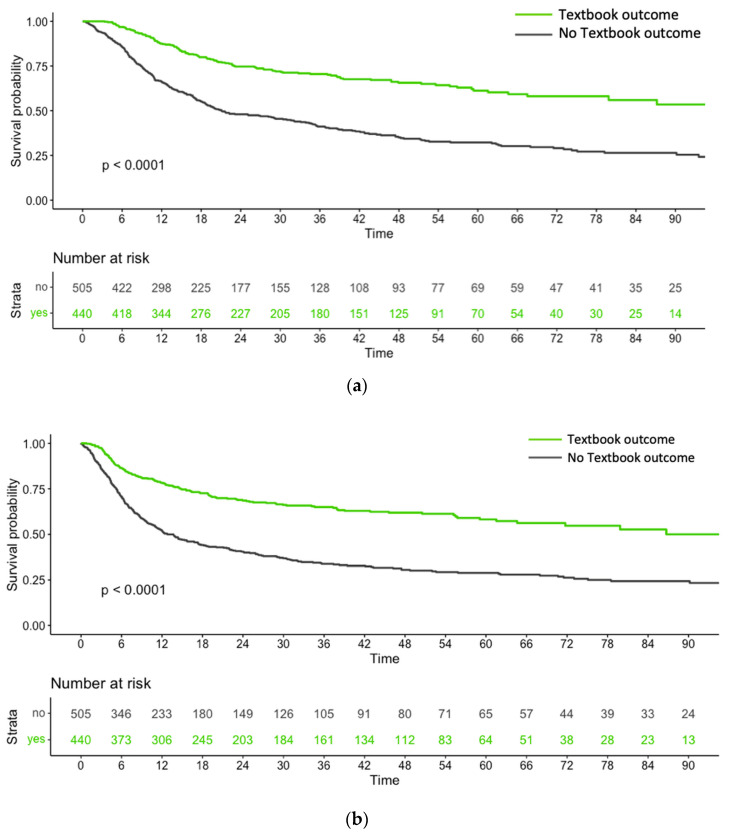
(**a**) Kaplan–Meier plots of overall survival (expressed in months) for patients who achieved textbook outcomes versus those who did not. (**b**) Kaplan–Meier plots of disease-free survival (expressed in months) for patients who achieved textbook outcomes versus those who did not.

The median OS was significantly longer in the TBO group at 109 months compared to the no-TBO group, which was 21 months (Figure 2a). This indicates an OS advantage of 88 months for the TBO group (*p* < 0.001). In addition, disease recurrences tended to be less frequent in the former group compared with the latter (42.7% versus 48.3%, respectively; *p* = 0.088). The median DFS was significantly longer in the TBO group at 87 months compared to the no-TBO group, which was 13 months (Figure 2b). This indicates a DFS advantage of 74 months for the TBO group (*p* < 0.001). 

#### 3.1.2. Univariate and Multivariable Predictors of TBO

In univariate analysis, nine factors were significantly associated with an increased likelihood of TBO, including a higher BMI, a lower CCI score, non-upper-third tumor location, adenocarcinoma histology, absence of preoperative radiotherapy, treatment at medical center A, undergoing an Ivor Lewis procedure, MIE with RE, and a minimally invasive abdominal approach (Table 2). However, after adjusting for potential confounders in multivariable analysis, only treatment with RE (OR = 1.527; 95% CI = 1.149–2.028) and the CCI score remained significant predictors. Specifically, a CCI score of 2 was associated with a lower likelihood of TBO (OR = 0.687, 95% CI = 0.483–0.977), as was a CCI score of 3 or higher (OR = 0.604, 95% CI = 0.399–0.915). Notably, the influence of the treatment center was no longer observed in the multivariable model.

#### 3.1.3. Associations of TBO with Different MIE Techniques (RE versus VATE)

Table 3 presents the baseline characteristics of the two patient groups, both prior to and following the application of IPTW. In the original cohort, patients who had undergone RE were characterized by a higher mean age and BMI, as well as a lower incidence of active smoking, in comparison to those who had received VATE. Significant differences were also evident with respect to several other variables, such as the distribution of clinical stages, the frequency of preoperative radiotherapy, and the treatment center. Regarding the surgical technique, a larger number of VATE patients underwent laparotomies and a higher proportion were subjected to the Ivor Lewis procedure. The implementation of IPTW resulted in achieving a satisfactory balance of covariates, as evidenced by all SMD values being below 0.1.

Table 4 provides a comprehensive summary of the impacts that the use of RE versus VATE had on each criterion defining TBO, both prior to and following the application of IPTW. The post-IPTW results indicate that patients who underwent RE experienced more favorable outcomes compared to those who received VATE in terms of the following specific parameters: (1) a higher percentage of patients achieved a lymph node yield of ≥15 (95.8% for RE versus 90.4% for VATE; *p* = 0.001); (2) a greater proportion of margin-negative resections was observed (94.2% for RE versus 88.6% for VATE; *p* = 0.005), and (3) a reduced incidence of major complications was noted (29.4% for RE versus 38.9% for VATE; *p* = 0.024). Additionally, the TBO rate post-IPTW was significantly higher for patients who received RE (53.3%) compared to those who underwent VATE (42.2%; *p* = 0.008)). 

## 4. Discussion

This is, to our knowledge, the first study to present detailed results about the achievement and prognostic significance of TBO in patients with EC who had undergone MIE. By adhering to the established definition of TBO and utilizing data from prominent surgical institutions, we were able to achieve a TBO rate of 43.2%, aligning with the highest standards reported in the literature (Table 5). Consistent with prior findings, the achievement of TBO was significantly associated with more favorable survival outcomes. Our findings are also significant as they demonstrate, for the first time, that within a cohort consisting entirely of MIE cases, the application of RE had the potential to enhance the total number of TBO. Moreover, the advantage of RE in improving TBO rates appeared robust, even after addressing potential selection bias through the implementation of IPTW. Given this evidence, we suggest that RE should be regarded as the surgical technique of choice for MIE procedures.

While VATE remains the most common technique for MIE, the adoption of robotic platforms has recently gained momentum [26,27]. RE offers numerous advantages, including a magnified, high-definition 3D visual field that ensures a stable, surgeon-controlled perspective, alongside improved ergonomics that enhance manual dexterity. These features are particularly advantageous for meticulous dissection within the narrow confines of the mediastinum. Recent meta-analyses have highlighted that RE is linked to reduced pulmonary complications and a greater yield of lymph nodes compared to VATE [28,29]. Upon examining the impacts of these surgical techniques on the criteria defining TBO, it was observed that patients undergoing RE experienced fewer major complications than those treated with VATE. More critically, RE was associated with a higher likelihood of achieving margin-negative resections and an increased count of harvested lymph nodes—two factors that are known to correlate with a more favorable prognosis [30,31,32,33]. Taken together, these findings suggest that RE not only potentially eases postoperative recovery, but also contributes to superior oncological outcomes. 

Notwithstanding the advantages over VATE in terms of TBO realization, a cost-effectiveness analysis of RE is imperative prior to its eventual assimilation into everyday surgical practice. Robotic surgery is indeed limited by high costs of acquisition and maintenance [34]. While market competition and the increasing adoption of robotic platforms could potentially reduce their costs in the future, effective training and accreditation are essential components. There is an urgent need to establish systematic training programs with the goal of shortening surgery proficiency gain curves. In this scenario, international robotic surgery societies—including the Upper GI International Robotic Association (UGIRA)—are expected to implement formal training programs for new generations of surgeons. Additionally, scientific societies should encourage the shared use of data among private and public stakeholders with the goal of evaluating the relative safety and efficacy of robotic operations in comparison to traditional procedures [35].

Although the potential benefits of RE observed in our investigation are encouraging, they must be weighed against the study’s inherent limitations. While multicenter study designs are recognized for their strengths, including the generalizability of findings and larger sample sizes, they can also introduce significant variability and error. Notably, the distinct characteristics of patients with EC between the West (with a higher prevalence of adenocarcinoma) and the East (where squamous cell carcinomas is predominant) led to major differences in the MIE approach. Center A favored the Ivor Lewis esophagectomy, whereas Center B preferred the McKeown procedure. Despite our efforts to account for these variables using IPTW, we must acknowledge the potential presence of unmeasured and residual confounding variables that could have influenced our findings. Notably, the decision to utilize RE in Center B was contingent upon a patient’s consent to undergo a partially insured robot-assisted procedure. This requirement could introduce a selection bias linked to the participants’ financial status. Secondly, it is important to note that RE was implemented after the introduction of VATE. This sequence of events leaves room for the possibility that the less favorable outcomes observed in the VATE group could be attributed to the initial learning curve associated with the introduction of this technique. Similarly, the superior results in the RE group might be influenced by the prior experience with VATE. Given these considerations, our results must be approached with caution and validated through future prospective randomized studies.

## 5. Conclusions

TBO was realized in 46.6% of the patients who underwent MIE for cancer. Patients with a lower CCI and those who received RE were more likely to achieve TBO.

## Figures and Tables

**Table 1:** General characteristics of patients with esophageal cancer who achieved textbook outcomes versus those who did not.

Variable	Entire Cohort	TBO(−)	TBO(+)	*p*
Number of patients (%)	945 (100)	505 (53.4)	440 (46.6)	
Age, years (mean (SD))	59.8 (10.49)	59.36 (10.36)	60.30 (10.61)	0.169
Sex				0.414
Female	104 (15.2)	60 (11.9)	44 (10.0)	
Male	841 (84.8)	445 (88.1)	396 (90.0)	
BMI, kg/m^2^ (mean (SD))	24.30 (8.36)	23.75 (4.43)	24.93 (11.24)	0.031
Charlson comorbidity index				0.043
0−1	647 (68.5)	328 (65)	319 (72.5)	
2	177 (18.7)	104 (20.6)	73 (16.6)	
≥3	121 (12.8)	73(14.4)	48 (10.9)	
Smoking				0.674
Never	387 (41)	201 (39.8)	186 (42.3)	
Quit > 30 days	127 (13.4)	67 (13.3)	60 (13.6)	
Active	431 (45.6)	237 (46.9)	194 (44.1)	
Tumor location				0.04
Upper third	117 (12.4)	72 (14.3)	45 (10.2)	
Middle third	265 (28)	157 (31.1)	108 (24.5)	
Lower third	563 (59.6)	276 (54.7)	287 (25.2)	
Histology				<0.001
Adenocarcinoma	351 (37.1)	161 (31.9)	190 (43.2)	
Squamous cell carcinoma	594 (62.9)	344 (68.1)	250 (56.8)	
cT-stage				0.232
cT1	102 (10.8)	57 (11.3)	45 (10.2)	
cT2−3	769 (81.4)	402 (79.6)	367 (83.4)	
cT4	74 (7.8)	46 (9.1)	28 (6.4)	
cN-stage				0.046
cN−	245 (25.9)	117 (23.2)	128 (29.1)	
cN+	700 (74.1)	388 (76.8)	312 (70.9)	
Preoperative radiotherapy				0.001
No	385 (40.7)	180 (35.6)	205 (46.6)	
Yes	560 (59.3)	325 (64.4)	235 (53.4)	
Preoperative chemotherapy				0.568
No	189 (20)	97 (19.2)	92 (20.9)	
Yes	756 (80)	408 (80.8)	348 (79.1)	
Medical center				0.001
Center A	426 (45.1)	201 (39.8)	225 (51.1)	
Center B	519 (54.9)	304 (60.2)	215 (48.9)	
Operative procedure				<0.001
Ivor Lewis	412 (43.6)	193 (38.2)	219 (49.8)	
McKeown	533 (56.4)	312 (61.8)	221 (60.2)	
Technique used for MIE				<0.001
VATE	537 (56.8)	319 (63.2)	218 (49.5)	
RE	408 (43.2)	186 (36.8)	222 (50.5)	
Abdominal part				0.01
Open surgery	122 (12.9)	79 (15.6)	43 (9.8)	
Minimally invasive surgery	823 (87.1)	426 (84.4)	397 (90.2)	

Abbreviations: TBO, textbook outcome; SD, standard deviation; BMI, body mass index; MIE, minimally invasive esophagectomy; VATE, video-assisted thoracoscopic esophagectomy; RE, robotic esophagectomy.

**Table 2:** Univariate and multivariable logistic regression analyses: predictors of textbook outcome.

	Univariate Analysis		Multivariable Analysis	
Variable	OR (95% CI)	*p*	OR (95% CI)	*p*
Age	1.009 (0.996–1.021)	0.169		
Sex				
Female	Reference	0.357		
Male	1.213 (0.804–1.832)			
BMI	1.036 (1.006–1.066)	0.019	1.016 (0.990–1.044)	0.235
Charlson comorbidity index				
0−1	Reference	0.044	Reference	0.015
2	0.722 (0.515–1.011)	0.058	0.687 (0.483–0.977)	0.037
≥3	0.676 (0.455–1.004)	0.052	0.604 (0.399–0.915)	0.017
Tumor location				
Upper third	Reference	0.004	Reference	0.660
Middle third	1.101 (0.705–1.719)	0.673	0.989 (0.626–1.561)	0.962
Lower third	1.664 (1.107–2.501)	0.014	1.168 (0.720–1.894)	0.530
Histology				
Squamous cell carcinoma	Reference	<0.001	Reference	0.917
Adenocarcinoma	1.624 (1.245–2.118)		0.977 (0.625–1.525)	
Preoperative radiotherapy				
No	Reference	0.001	Reference	0.053
Yes	0.635 (0.489–0.824)		0.729 (0.530–1.004)	
Preoperative chemotherapy				
No	Reference	0.514		
Yes	0.899 (0.654–1.237)			
Medical center				
Center A	Reference	<0.001	Reference	0.878
Center B	0.632 (0.488–0.818)		1.090 (0.361–3.293)	
Operative procedure				
Ivor Lewis	Reference	<0.001	Reference	0.780
McKeown	0.624 (0.482–0.809)		0.851 (0.274–2.641)	
Technique used for MIE				
VATE	Reference	<0.001	Reference	0.004
RE	1.747 (1.347–2.265)		1.527 (1.149–2.028)	
Abdominal part				
Open surgery	Reference	0.008	Reference	0.357
Minimally invasive surgery	1.712 (1.153–2.544)		1.227 (0.794–1.897)	

Abbreviations: OR, odds ratio; CI, confidence interval; BMI, body mass index; MIE, minimally invasive esophagectomy; VATE, video-assisted thoracoscopic esophagectomy; RE, robotic esophagectomy.

**Table 3:** General characteristics of the two study groups before and after the application of inverse probability of treatment weighting.

Characteristic	Original Cohort			IPTW Cohort		
	RE (*n* = 408)	VATE (*n* = 537)	SMD	RE	VATE	SMD
Men, *n* (%)	360 (88.2%)	481 (89.6%)	0.043	90.0%	89.2%	0.028
Age, years	61.76 ± 10.835	58.31 ± 9.968	0.331	59.26 ± 11.02	59.93 ± 10.46	0.063
Body mass index, kg/m^2^	25.15 ± 11.686	23.66 ± 4.297	0.169	24.30 ± 9.97	24.27 ± 4.52	0.004
Smoking, *n* (%)			0.506			0.079
No	222 (54.41%)	165 (30.73%)		39.63%	41.36%	
Quit > 30 days	50 (12.25%)	77 (14.34%)		11.28%	13.12%	
Active smoker	136 (33.34)	295 (54.93%)		49.09%	45.52%	
CCI			0.110			0.101
0–1	280 (68.63%)	367 (68.34%)		63.31%	68.12%	
2	69 (16.91%)	108 (20.11%)		21.74%	18.86%	
3	59 (14.46%)	62 (11.55%)		14.95%	13.02%	
Histology			0.575			0.003
Squamous cell carcinoma	194 (47.55%)	400 (74.49%)		62.07%	62.20%	
Adenocarcinoma	214 (52.45)	137 (25.51%)		37.93%	37.80%	
Clinical AJCC stage			0.198			0.080
I	38 (9.31%)	54 (10.06%)		11.34%	10.04%	
II	120 (29.41%)	118 (21.97%)		26.02%	25.62%	
III	191 (46.81%)	259 (48.23%)		48.15%	47.18%	
IV	59 (14.47%)	106 (19.74)		14.49%	17.16%	
Preop Radiotherapy	214 (52.45%)	346 (64.43%)	0.245	58.05%	57.60%	0.009
Preop Chemotherapy	328 (80.39%)	428 (79.70%)	0.017	78.17%	78.35%	0.004
Center			0.727			0.015
A	263 (64.46%)	163 (30.35%)		44.44%	45.21%	
B	145 (35.54%)	374 (69.65%)		55.56%	54.79%	
Abdominal surgical technique			0.631			0.034
Laparoscopy	400 (98.04%)	423 (78.77%)		85.92%	87.08%	
Laparotomy	8 (1.96%)	114 (21.23%)		14.08%	12.92%	
Type of resection			0.698			0.016
Ivor Lewis	254 (62.25%)	158 (29.42%)		42.97%	43.76%	
McKeown	154 (37.75%)	379 (70.58%)		57.03%	56.24%	

Data are expressed as means and standard deviations or counts and percentages, as appropriate. Abbreviations: IPTW, inverse probability of treatment weighting; RE, robotic esophagectomy; VATE, video-assisted thoracoscopic esophagectomy; SMD, standardized mean difference; CCI, Charlson comorbidity index; AJCC, American Joint Committee on Cancer.

**Table 4:** Overall incidence and severity of complications before and after the application of inverse probability of treatment weighting.

	Before IPTW		*p*	After IPTW		*p*
*n* (%)	RE	VATE		RE	VATE	
TBO items						
(1) No intraop complication	98.5%	96.5%	0.05	98.7%	96.8%	0.071
(2) Lymph node yield ≥ 15	388 (95.1%)	475 (88.5%)	<0.001	95.8%	90.4%	0.001
(3) LOS ≤ 21 days	81.9%	75%	0.012	78.0%	76.6%	0.729
(4) No need of reintervention	78.2%	65%	<0.001	74.2%	67.0%	0.086
(5) Margin-negative resection	93.9%	87.2%	0.001	94.2%	88.6%	0.005
(6) No readmission to an ICU	92.2%	90.9%	0.493	91.0%	91.9%	0.692
(7) No readmissions within 30 days from discharge	87.5%	84.9%	0.256	88.6%	84.7%	0.098
(8) N0 major complications	73.3%	60%	<0.001	70.6%	61.1%	0.024
(9) No 90-day postoperative mortality	97.1%	95.5%	0.224	97.6%	95.2%	0.056
Overall TBO rates	54.4%	40.6%	<0.001	53.3%	42.2%	0.008
Non-TBO items						
(1) Number of harvested nodes (mean (SD))	32.73 ± 13.343	28.80 ± 13.086	<0.001	33.33 ± 14.07	29.62 ± 12.78	0.001
(2) LOS, days (mean (SD))	17.06 ± 13.381	21.47 ± 19.037	<0.001	17.77 ± 13.11	20.87 ± 19.57	0.017
(3) Blood loss, mL (mean (SD))	132.43 ± 94.556	170.82 ± 187.386	<0.001	120.92 ± 89.49	169.31 ± 170.16	<0.001
(4) Total operation time, min (mean (SD))						
Thoracic	189.63 ± 54.02	226.32 ± 727.29	0.312	176.35 ± 51.31	218.96 ± 637.59	0.147
Abdomen	139.42 ± 42.43	133.58 ± 53.95	0.177	145.34 ± 44.41	126.59 ± 49.44	<0.001

Abbreviations: IPTW, inverse probability of treatment weighting; RE, robotic esophagectomy; VATE, video-assisted thoracoscopic esophagectomy; TBO, textbook outcome; LOS, length of stay.

**Table 5:** Summary of published studies focusing on the achievement of textbook outcomes following esophagectomy.

Authors (Year of Publication)	Study Design	Sample Size	Minimally Invasive Surgery, *n* (%)	RE, *n* (%)	TBO Rate	Survival Impact
Busweiler et al. (2017) [11]	Nationwide study	2748	1347 (49%)	N/A	29.7%	N/A
Van der Werf et al. (2019) [12]	Nationwide study	4414	2595 (58.8%)	N/A	33%	Yes
Bolger et al. (2021) [20]	Single-center study	269	130 (48.3%)	N/A	32.3%	Yes
Kalff et al. (2021) [7]	Two-center study	1065	676 (63.5%)	N/A	30.7%	Yes
Current study	Two-center study	945	945 (100%)	408 (43.2%)	46.6%	Yes

Abbreviations: RE, robotic esophagectomy; TBO, textbook outcome; N/A, not available.

## Data Availability

The data presented in this study are contained within the article.

## References

[1] van Hagen P., Hulshof M.C., van Lanschot J.J., Steyerberg E.W., van Berge Henegouwen M.I., Wijnhoven B.P., Richel D.J., Nieuwenhuijzen G.A., Hospers G.A., Bonenkamp J.J. (2012). Preoperative chemoradiotherapy for esophageal or junctional cancer. N. Engl. J. Med..

[2] Yang H., Liu H., Chen Y., Zhu C., Fang W., Yu Z., Mao W., Xiang J., Han Y., Chen Z. (2018). Neoadjuvant chemoradiotherapy followed by surgery versus surgery alone for locally advanced squamous cell carcinoma of the esophagus (NEOCRTEC5010): A phase III multicenter, randomized, open-label clinical trial. J. Clin. Oncol..

[3] Bhagat R., Bronsert M.R., Juarez-Colunga E., Weyant M.J., Mitchell J.D., Glebova N.O., Henderson W.G., Fullerton D., Meguid R.A. (2018). Postoperative complications drive unplanned readmissions after esophagectomy for cancer. Ann. Thorac. Surg..

[4] In H., Palis B.E., Merkow R.P., Posner M.C., Ferguson M.K., Winchester D.P., Pezzi C.M. (2016). Doubling of 30-day mortality by 90 days after esophagectomy. Ann. Surg..

[5] D’Journo X.B., Boulate D., Fourdrain A., Loundou A., van Berge Henegouwen M.I., Gisbertz S.S., O’Neill J.R., Hoelscher A., Piessen G., Van Lanschot J. (2021). Risk prediction model of 90-day mortality after esophagectomy for cancer. JAMA Surg..

[6] Kolfschoten N.E., Kievit J., Gooiker G.A., van Leersum N.J., Snijders H.S., Eddes E.H., Tollenaar R.A., Wouters M.W., Marang-van de Mheen P.J. (2013). Focusing on desired outcomes of care after colon cancer resections; hospital variations in ‘textbook outcome’. Eur. J. Surg. Oncol..

[7] Kalff M.C., Vesseur I., Eshuis W.J., Heineman D.J., Daams F., van der Peet D.L., van Berge Henegouwen M.I., Gisbertz S.S. (2021). The association of textbook outcome and long-term survival after esophagectomy for esophageal cancer. Ann. Thorac. Surg..

[8] Kulshrestha S., Bunn C., Patel P.M., Sweigert P.J., Eguia E., Pawlik T.M., Baker M.S. (2020). Textbook oncologic outcome is associated with increased overall survival after esophagectomy. Surgery.

[9] Dal Cero M., Román M., Grande L., Yarnoz C., Estremiana F., Gantxegi A., Codony C., Gobbini Y., Garsot E., Momblan D. (2022). Textbook outcome and survival after gastric cancer resection with curative intent: A population-based analysis. Eur. J. Surg. Oncol..

[10] Kalagara R., Norain A., Chang Y.-H., Stucky C.-C., Wasif N. (2022). Association of textbook outcome and surgical case volume with long-term survival in patients undergoing surgical resection for pancreatic cancer. J. Am. Coll. Surg..

[11] Busweiler L., Schouwenburg M., van Berge Henegouwen M., Kolfschoten N., de Jong P., Rozema T., Wijnhoven B., van Hillegersberg R., Wouters M., van Sandick J. (2017). Textbook outcome as a composite measure in oesophagogastric cancer surgery. J. Br. Surg..

[12] Van Der Werf L.R., Wijnhoven B.P., Fransen L.F., van Sandick J.W., Nieuwenhuijzen G.A., Busweiler L.A., van Hillegersberg R., Wouters M.W., Luyer M.D., van Berge Henegouwen M.I. (2019). A national cohort study evaluating the association between short-term outcomes and long-term survival after esophageal and gastric cancer surgery. Ann. Surg..

[13] de Groot E., Goense L., Kingma B., Haverkamp L., Ruurda J., van Hillegersberg R. (2023). Trends in surgical techniques for the treatment of esophageal and gastroesophageal junction cancer: The 2022 update. Dis. Esophagus.

[14] Haverkamp L., Seesing M., Ruurda J., Boone J. (2017). Worldwide trends in surgical techniques in the treatment of esophageal and gastroesophageal junction cancer. Dis. Esophagus Off. J. Int. Soc. Dis. Esophagus.

[15] Dyas A.R., Stuart C.M., Bronsert M.R., Schulick R.D., McCarter M.D., Meguid R.A. (2023). Minimally invasive surgery is associated with decreased postoperative complications after esophagectomy. J. Thorac. Cardiovasc. Surg..

[16] Yerokun B.A., Sun Z., Yang C.-F.J., Gulack B.C., Speicher P.J., Adam M.A., D’Amico T.A., Onaitis M.W., Harpole D.H., Berry M.F. (2016). Minimally invasive versus open esophagectomy for esophageal cancer: A population-based analysis. Ann. Thorac. Surg..

[17] Ising M.S., Smith S.A., Trivedi J.R., Martin R.C., Phillips P., Van Berkel V., Fox M.P. (2023). Minimally invasive esophagectomy is associated with superior survival compared to open surgery. Am. Surg..

[18] Mariette C., Markar S., Dabakuyo-Yonli T.S., Meunier B., Pezet D., Collet D., D’journo X.B., Brigand C., Perniceni T., Carrere N. (2020). Health-related quality of life following hybrid minimally invasive versus open esophagectomy for patients with esophageal cancer, analysis of a multicenter, open-label, randomized phase III controlled trial: The MIRO trial. Ann. Surg..

[19] Mariette C., Markar S.R., Dabakuyo-Yonli T.S., Meunier B., Pezet D., Collet D., D’Journo X.B., Brigand C., Perniceni T., Carrere N. (2019). Hybrid Minimally Invasive Esophagectomy for Esophageal Cancer. N. Engl. J. Med..

[20] Bolger J.C., Al Azzawi M., Whooley J., Bolger E.M., Trench L., Allen J., Kelly M.E., Brosnan C., Arumugasamy M., Robb W.B. (2021). Surgery by a minimally invasive approach is associated with improved textbook outcomes in oesophageal and gastric cancer. Eur. J. Surg. Oncol..

[21] Charlson M., Szatrowski T.P., Peterson J., Gold J. (1994). Validation of a combined comorbidity index. J. Clin. Epidemiol..

[22] Reynolds J.V., Donlon N., Elliott J.A., Donohoe C., Ravi N., Kuppusamy M.K., Low D.E. (2021). Comparison of Esophagectomy outcomes between a National Center, a National Audit Collaborative, and an International database using the Esophageal Complications Consensus Group (ECCG) standardized definitions. Dis. Esophagus.

[23] Dindo D., Demartines N., Clavien P.A. (2004). Classification of surgical complications: A new proposal with evaluation in a cohort of 6336 patients and results of a survey. Ann. Surg..

[24] Desai R.J., Franklin J.M. (2019). Alternative approaches for confounding adjustment in observational studies using weighting based on the propensity score: A primer for practitioners. BMJ.

[25] Heinze G., Jüni P. (2011). An overview of the objectives of and the approaches to propensity score analyses. Eur. Heart J..

[26] Kamel M.K., Sholi A.N., Rahouma M., Harrison S.W., Lee B., Stiles B.M., Altorki N.K., Port J.L. (2021). National trends and perioperative outcomes of robotic oesophagectomy following induction chemoradiation therapy: A National Cancer Database propensity-matched analysis. Eur. J. Cardio-Thorac. Surg..

[27] Kingma B.F., Grimminger P.P., van der Sluis P.C., van Det M.J., Kouwenhoven E.A., Chao Y.-K., Tsai C.-Y., Fuchs H.F., Bruns C.J., Sarkaria I.S. (2022). Worldwide techniques and outcomes in robot-assisted minimally invasive esophagectomy (RAMIE): Results from the multicenter international registry. Ann. Surg..

[28] Manigrasso M., Vertaldi S., Marello A., Antoniou S.A., Francis N.K., De Palma G.D., Milone M. (2021). Robotic esophagectomy. A systematic review with meta-analysis of clinical outcomes. J. Pers. Med..

[29] Zhang Y., Dong D., Cao Y., Huang M., Li J., Zhang J., Lin J., Sarkaria I.S., Toni L., David R. (2023). Robotic versus conventional minimally invasive esophagectomy for esophageal cancer: A meta-analysis. Ann. Surg..

[30] Henckens S.P., Hagens E.R., van Berge Henegouwen M.I., Meijer S.L., Eshuis W.J., Gisbertz S.S. (2023). Impact of increasing lymph node yield on staging, morbidity and survival after esophagectomy for esophageal adenocarcinoma. Eur. J. Surg. Oncol..

[31] Ho H.-J., Chen H.-S., Hung W.-H., Hsu P.-K., Wu S.-C., Chen H.-C., Wang B.-Y. (2018). Survival impact of total resected lymph nodes in esophageal cancer patients with and without neoadjuvant chemoradiation. Ann. Surg. Oncol..

[32] Gu Y.M., Yang Y.S., Kong W.L., Shang Q.X., Zhang H.L., Wang W.P., Yuan Y., Che G.W., Chen L.Q. (2022). Effect of circumferential resection margin status on survival and recurrence in esophageal squamous cell carcinoma with neoadjuvant chemoradiotherapy. Front. Oncol..

[33] Hollertz P., Lindblad M., Sandström P., Halldestam I., Edholm D. (2021). Outcome of microscopically non-radical oesophagectomy for oesophageal and oesophagogastric junctional cancer: Nationwide cohort study. BJS Open.

[34] Rebecchi F., Ugliono E., Allaix M.E., Morino M. (2023). Why pay more for robot in esophageal cancer surgery?. Updates Surg..

[35] Kingma B.F., Hadzijusufovic E., Van der Sluis P.C., Bano E., Lang H., Ruurda J.P., van Hillegersberg R., Grimminger P.P. (2020). A structured training pathway to implement robot-assisted minimally invasive esophagectomy: The learning curve results from a high-volume center. Dis. Esophagus.

